# Percutaneous nephrostomy in Ureteropelvic junction obstruction with poorly functioning kidney: Is it still pertinent in adults?

**DOI:** 10.5152/tud.2022.22050

**Published:** 2022-05-01

**Authors:** Uday Pratap Singh, Shitangsu Kakoti, Sanjoy Kumar Sureka, Nayab Danish, Abhay Kumar, Zain Tamboli, Madhur Anand, Aneesh Srivastava

**Affiliations:** 1 Department of Urology and Renal Transplantation, Sanjay Gandhi Postgraduate Institute of Medical Sciences, Lucknow, India; 2 Department of Urology, Maharani Laxmi Bai Medical College, Jhansi, India

**Keywords:** Nephrostomy, percutaneous, ureteral obstruction, hydronephrosis, kidney

## Abstract

**Objective:**

To determine the pertinence of percutaneous nephrostomy drainage in adult patients of primary ureteropelvic junction obstruction with poorly functioning kidneys (<20% split renal function).

**Material and methods:**

Clinical records of all patients with primary ureteropelvic junction obstruction with poorly functioning kidneys who underwent percutaneous nephrostomy drainage in our institute between February 2015 and January 2020 were retrospectively reviewed. The patients were divided into 4 groups according to their split renal function obtained from the Tc-99m ethylenedicysteine diuretic renogram. Group I consisted of all patients having split renal function ≤5%, group II with split renal function 6-10%, group III with split renal function 11-15%, and finally group IV with split renal function 16-20%. Those patients in whom split renal function was improved by >10% and had daily percutaneous nephrostomy output >400 mL, underwent pyeloplasty and the rest underwent nephrectomy.

**Results:**

Seventy-two patients were studied, out of which 5 were in group I, 20 in groups II and III each, and 27 in group IV. The mean age of presentation was 34.4 ± 14 years. The split renal function improvement of >10% was seen in 55 patients (76.4%) after percutaneous nephrostomy drainage (*P* < .05). Pyeloplasty was done in 40 patients (55.6%) and nephrectomy was done in 32 patients (44.4%).

**Conclusion:**

In conclusion, we recommend the use of a Tc-99m ethylenedicysteine scan for estimation of split renal function during the initial presentation in every patient followed by reconstructive surgery if split renal function is above 15% and nephrectomy if it is below 5%. The trial of percutaneous nephrostomy is pertinent if split renal function is between 6% and 15%.

**Main Points**

- Literature does not define distinct cut-off values of split renal function (SRF) in an adult patient with ureteropelvic junction obstruction where a percutaneous nephrostomy (PCN) would be ideally pertinent.
- We found that if SRF is above 15%, pyeloplasty is an ideal choice.
- If SRF is below 5%, the patient should undergo a nephrectomy.
- A trial of PCN is pertinent if SRF is between 6% to 15%.

## Introduction

Ureteropelvic junction (UPJ) obstruction in adults leads to impaired urinary drainage and in some cases responsible for renal cortical atrophy and poor function. The management of UPJ obstruction with a relatively normal functioning kidney is pyeloplasty (PP), but for a poorly functioning kidney (PFK, split renal function (SRF) <20%), it is still debatable.^1–4^ This is because the definition of a PFK includes a wide spectrum of renal functions which are assessed by different methods. Moreover, the renal radionucleotide scans that are mostly used worldwide for determining renal function are not always reliable for severely obstructed kidneys.^3^ In such cases, a trial of percutaneous nephrostomy (PCN) to obtain a near accurate glomerular filtration rate (GFR) value and to a certain degree predict recovery of renal function has been described^2,5^ as it has been realized that those kidneys who recover function following PCN are likely to recover after PP.^5^ However, PCN has its inherent morbidity and may also lead to delay in the definitive management. The survey of the existing literature does not reveal any distinct cut-off values of SRF as determined by the nuclear scan where the PCN would be ideally indicated or pertinent. The objective of this study is to determine the valid role of PCN in these scenarios.

We therefore retrospectively analyzed our data in patients with UPJ obstruction with PFK by stratifying them into 4 groups, based on their SRF, and tried to find out the impact of PCN in each group in determining the near accurate renal function and renal function recovery so that we can either opt or avoid PCN judiciously thereby defining its relevance.

## Material and Methods

This is a retrospective study done in a tertiary care center from data of patients admitted between February 2015 and January 2020 after taking informed consents as well as approval from the institutional ethical committee of Sanjay Gandhi Postgraduate Institute of Medical Sciences (2021-221-MCh-EXP-42). Around 350 patients underwent PCN drainage in this center in the last 5 years for a variety of reasons like calculus disease (70%), UPJ obstruction (20.6%), genitourinary tuberculosis (5%), and others (5%). Percutaneous nephrostomy drainage is routinely performed for UPJ obstruction with poorly functioning kidneys before any definitive surgery. However, around 158 patients underwent PP directly without prior PCN drainage. We meticulously reviewed our database and handpicked those patients who presented to us with a unilateral primary UPJ obstruction with SRF ≤20% and underwent PCN drainage to include them in this study. All those patients who were <18 years of age, had bilateral or secondary UPJ obstruction due to stones, strictures, other causes of upper tract dilatations like Vesicoureteral reflux (VUR), congenital anomalies like duplex system, small kidney or single functioning kidney, or PCN duration of <4 weeks were excluded from this study. Data were collected regarding clinical presentations and findings, pre-procedure investigations, post-procedural complications, post-procedural investigation findings, follow-up, and outcome. The findings of ultrasonography of kidney, ureter, and bladder (USG KUB) and diuretic renal scintigraphy done before and after PCN drainage were noted. The patients were divided arbitrarily into 4 groups according to their SRF obtained from an ethylenedicysteine (EC) diuretic renogram done before PCN drainage. Group I consist of all patients having SRF ≤5%, Group II with SRF 6-10%, Group III with SRF 11-15%, and finally group IV with SRF 16-20%. Data were entered separately for each group and later compared with appropriate statistical methods.

The radionucleotide study routinely done was a Tc-99m EC scan. In the EC scan, GFR was calculated from effective renal plasma flow (eRPF) using the following formula, GFR = eRPF/3.5.^3^

Percutaneous nephrostomy was done under combined ultrasonographic and fluoroscopic guidance by an uroradiologist under local anesthesia. The patients were put in a prone position on the fluoroscopy table and a trans retroperitoneal approach was employed. Mostly posterior calyx of the lower or mid pole was preferred.^6^ Daily PCN output was measured and documented.

Glomerular filtration rate estimation from PCN creatinine clearance (ccGFR) was also done in all cases after stabilization of PCN output usually after 4-5 days.^3^ A repeat EC scan was performed in every patient after 4-6 weeks of PCN placement. Those whose SRF and GFR improved by >10% of baseline function and daily PCN output was >400 mL after stabilization underwent PP and the rest underwent nephrectomy (Nx). All patients were followed up till they underwent either PP or nephrectomy. Those who underwent PP were further followed up at 3, 6, and 12 months and then annually with clinical examination, ultrasonography, and diuretic renal scan. The same criteria of renal function improvement were applied here too.

The statistical analysis was done with the help of IBM SPSS Statistics for Windows, version 26 (IBM Corp., Armonk, NY, USA). For continuous data one-way analysis of variance test or Kruskal–Wallis H test was used. The comparison was done by paired *t*-test or Wilcoxon signed-rank test and correlation analysis was done using the Pearson correlation coefficient. For all categorical data, chi-squared test or Fisher’s exact test was utilized.

## Results

A total of 72 patients were eligible for this study out of which 5 were in group I (SRF ≤5%), 20 in group II (SRF 6-10%) and III (SRF11-15%) each, and 27 in group IV (SRF 16-20%). The mean age of presentation was 34.4 ± 14 years and males were the predominant population (65.3%). The most common presenting symptom was pain (68%) and the most common side involved was the left side (61%). Other demographic findings are mentioned in Table 1. The average PCN output was 757 mL/day after stabilization which was statistically significant when compared among different groups (*P* = .00) and the mean PCN duration was 8.1 ± 4.1 weeks. During the PCN procedure, 5 patients suffered from the perirenal hematoma, and 2 patients had pleural breach which was treated conservatively. Hematuria, fever, and PCN catheter displacement were the post-procedural complications encountered out of which only PCN displacement required active intervention in the form of catheter repositioning or replacement. A PCN displacement was diagnosed when patients complain of low PCN output and confirmed by a nephrostogram. The ccGFR that was calculated in all patients had a mean of 8.3 ± 4.1 mL/min. A strong correlation was also seen between ccGFR and the pre-PCN GFR estimated from a diuretic renal scan with Pearson correlation coefficient = 0.783 and *P* < .001. The mean length of hospital stays after PCN was 2.4 ± 0.7 days and the median follow-up time was 13.8 months for post-PP patients. (Table 1)

The SRF and GFR improvement of >10% was seen in 51 patients (70.8%) after PCN drainage, out of which 0 patients were in group I, 10 in group II, 14 in group III, and 27 were in group IV (*P* < .05). (Table 2). 

Further comparing the means of SRF and GFR of the diseased kidney before and after the PCN drainage, we had found a statistically significant improvement in each parameter (*P* < .05). The mean SRF and GFR increased from 13.2 ± 5% to 17.3 ± 8.8% and 9.2 ± 4.6 mL/min to 12 ± 7.2 mL/min, respectively. 

Renal function improvement was seen in 51 patients, but 11 patients had PCN output of <400 mL/day after stabilization. So, finally, reconstructive surgery in the form of PP was done in 40 patients (55.6%), and nephrectomy was done in 32 patients (44.4%). 

A follow-up EC scan was done at 1 year in patients who underwent PP showed a mean SRF of 18.9 ± 5.8%. Out of 40 patients, SRF was improved in 18, stable in 12, and deteriorated in 10 patients. Among the patients with deteriorated renal function, 2 underwent nephrectomy, 3 refused any intervention, and 5 were lost to follow-up after 3 years.

## Discussion

To define poorly functioning kidneys, most researchers worldwide either use an SRF obtained from a diuretic renal scintigraphy or a PCN creatinine clearance. In a severely hydronephrotic kidney, often PCS gets included in the region of interest (ROI) while performing nuclear scans which ultimately leads to an overestimation of SRF or GFR.^3,7,8^ Moreover, only 10% of creatinine is secreted by tubular secretion so GFR calculated from creatinine clearance also overestimates it.^3^ Since most of the methods of renal function estimation have their flaws and there is no consensus in the literature for a specific cut-off value of SRF for undergoing nephrectomy, surgeons over the world prefer to use their institutional protocol while deciding the management. Clinical guidelines usually recommend tubular agents (EC scan, etc.) for diuretic renal scintigraphy over glomerular agents like Diethylenetriamine pentaacetate (DTPA) because of their larger volume distribution, better extraction efficiency, and kidney background ratio.^9–11^ Among the tubular agents, EC scan is more readily available in our country; therefore, considering all the benefits, we in our practice used EC scan as an initial modality to calculate SRF. However, the EC scan fails to measure GFR directly instead, it gives an eRPF value, from which GFR is calculated using the following formula.

GFR = eRPF × filtration fraction (FF)/extraction ratio (ER).^3^

The FF of humans is 0.2^12^ and ER of EC is 0.7.^13^ Thus, the final formula appears as GFR = eRPF/3.5.

Once the SRF is measured with an EC scan, it is followed by either PCN or definitive treatment. Besides, before opting for a nephrectomy nobody prefers to rely on a single test. It is worth mentioning that, PP in PFK is also associated with a longer follow-up time and increased financial burden, and if a complication occurs it will have higher morbidity than with nephrectomy and may require additional surgery.^14^

Theoretically, in USG KUB if renal echogenicity and cortical thickness are normal, corticomedullary differentiation (CMD) is maintained and there is no evidence of renal cysts it indicates better recovery.^15^ But when we compared our data, we hardly found any significant impact of these parameters in ultimate decision-making for renal salvageability. Among our patients out of 72, 56 had a parenchymal thickness of less than 10 mm (28-PP; 28-Nx), 25 had lost CMD (19-PP; 6-Nx), 59 had raised echogenicity (32-PP; 27-Nx), and 5 had renal cysts (2-PP; 3-Nx). Likewise, many authors also believe that these preoperative parameters are not strong predictors of postoperative renal function^14^ so a trial of diversion may be beneficial.^5^ Percutaneous nephrostomy and double J stent (DJS) are the feasible options available but with their limitations. Double J stent has a lower quality of life due to lower urinary tract symptoms whereas PCN has comparatively longer hospital stays, a higher rate of sepsis, and more anxiety issues.^16,17^ The advantage of PCN is that it can directly monitor the urine output and also provide us samples for urinalysis from the diseased kidney. We found 20 patients with infected urine of which 10 each underwent PP and nephrectomy respectively. GFR estimated from PCN creatinine clearance can also serve as an adjunct to the nuclear scan methods as its results are comparable to GFR obtained from DTPA scan or that calculated from eRPF of EC scan. In the case of DJS, sometimes a small amount of urine drains into the ureter and underestimates GFR^3^ Proponent of PCN describe it as the best method to see potential renal recovery in PFK.^2,5^ While others oppose it by saying, PCN cannot improve renal function and their outcome if SRF <15 in adults.^18^

In this study, we have found that like several other studies, the clinical characteristics of a patient with UPJ obstruction with PFK rarely exhibit significant differences when compared among different groups and cannot be considered as a predictor of renal function recovery.^19^ However, following PCN drainage, our study showed improved GFR and SRF in 70.8% of cases. Although there is a discrepancy between absolute values of pre-PCN SRF and GFR possibly due to the condition of the contralateral kidney in some cases which affects SRF, we have found a strong correlation between the 2 parameters (Pearson correlation coefficient (*r*) = 0.967 and *P* < .001) which allowed us to use them as a surrogate of one another. Among the groups, greater improvement of function was seen with group IV, followed by group III and then group II, which implies better functioning kidneys recover slightly better after PCN drainage. 

Besides a few minor postprocedural complications and morbidity associated with carrying an indwelling catheter, PCN helps in multiple ways. First, it relieves the chronic obstruction and thereby allows the kidney to recover function, second, it helps to measure urine output directly from the diseased kidney and thereby calculate creatinine clearance, third, it also helps nuclear scans to measure true renal function by effectively excluding PCS from ROI, and last but not the least in rare cases of subclinical infection with a dubious radiological diagnosis it aptly prevents potential postoperative sepsis. In their respective papers, many authors acknowledged a >10% increase in SRF and PCN output of >400 mL/24 hours as evidence of salvageable renal function which can be considered for reconstructive surgery.^1,2,18^ We too have also noticed similar findings in group IV (SRF 16-20%) patients who had daily PCN drainage of more than 400 mL and a few patients of group II and III whose SRF improved by 10% and had daily PCN output of >400ml underwent PP. Thus, by comparing the renal function before and after PCN and measuring daily PCN drainage we can safely predict the accurate renal function in a particular patient and thereby decide on nephrectomy on a patient-to-patient basis. Moreover, most of these studies are done on pediatric age groups^5,20–23^ and only a few in adults^1,2,24^ that also only describe the efficacy of trial of PCN in renal salvageability while our study provides an in-depth analysis among different SRF groups and additionally disproves the need of PCN in the kidney of SRF <5% and >15%.

Although radionucleotide scan is the most popular method for GFR estimation worldwide due to its non-invasiveness, easy availability, and minimal adverse effects, questions are still raised over its applicability in cases of grossly hydronephrotic poorly functioning kidneys. Percutaneous nephrostomy can be employed easily in this setting and can effectively predict outcomes and avoid unnecessary surgery. The GFR estimated from creatinine clearance of PCN drainage can act as an adjunct to radionucleotide scans whenever there is a discrepancy between clinical and radiological findings or even substitute nuclear scans in instances of their unavailability or contraindication. However, PCN use should be judicious enough to prevent unwarranted morbidity and anxiety among patients who are least benefitted from it.

**Figure 1.**
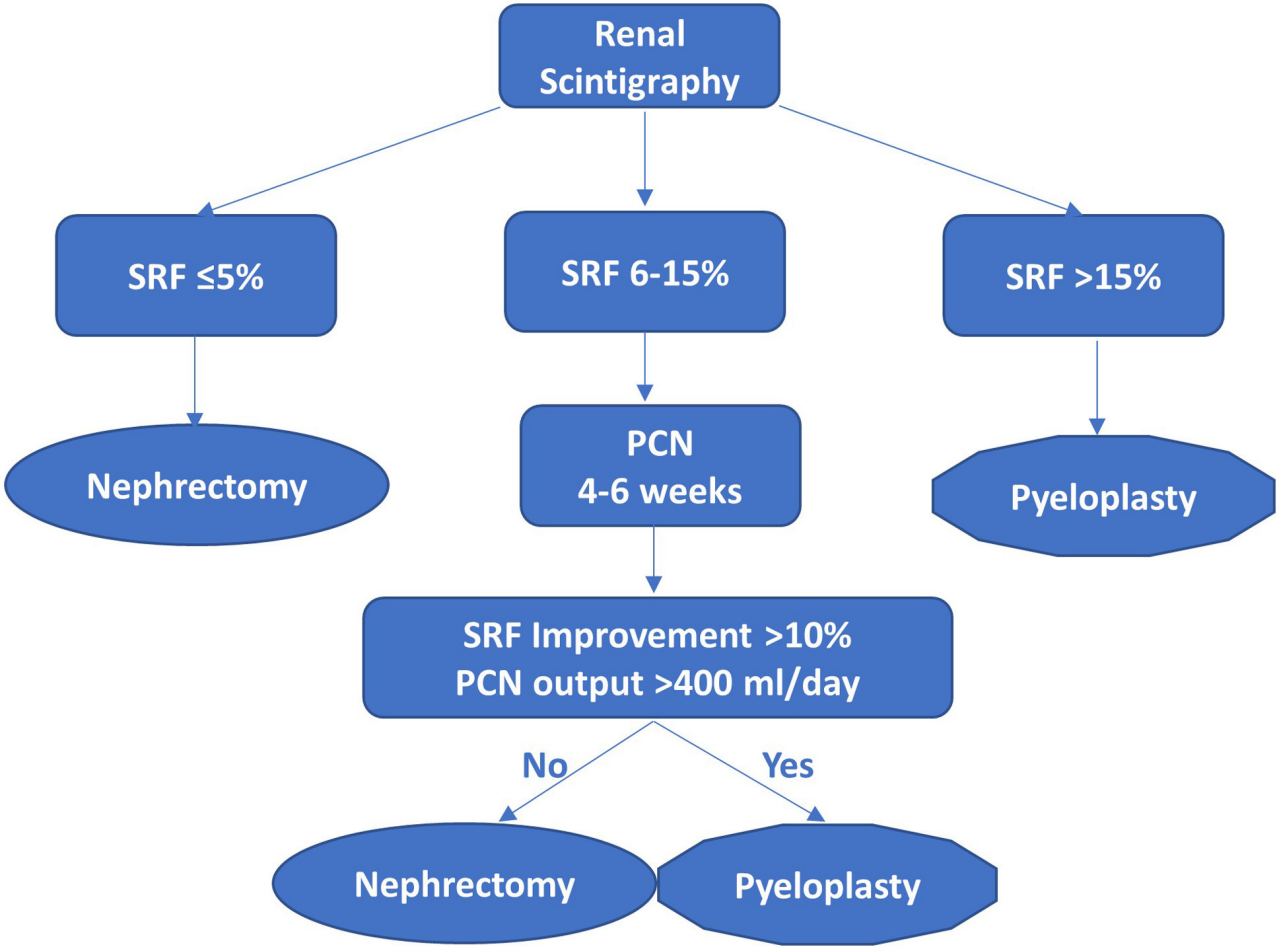
Conclusion from our study.

In conclusion**, **we recommend the use of renal scintigraphy for the estimation of SRF during an initial presentation in every patient with UPJ obstruction and PFK. Pyeloplasty should be the choice if SRF is above 15% and nephrectomy if it is below 5%. The trial of PCN is pertinent if SRF is between 6% and 15% (Figure 1).

### Limitations

This is a retrospective study done in a single institution with a smaller sample size and requires external validation.

## Figures and Tables

**Table 1.** Clinical Characteristics and Demography

**Variables**	**Total**		**Group I-(Split Renal Function 1-5%)**	**Group II-(Split Renal Function 6-10%)**	**Group III-(Split Renal Function 11-15%)**	**Group IV-(Split Renal Function 16-20%)**	*P*
Total (n)	72		5	20	20	27	
Gender							
Male	47 (65.3%)		3	15	12	17	.766
Female	25 (34.7%)		2	5	8	10
Age (mean ± SD)	34.4 ± 14		29.2 ± 4.9	33.5 ± 15	37.9 ± 15.4	33.6 ± 12.9	.545
Disease side							
Left	44 (61.1%)		3	15	12	14	.46
Right	28 (38.9%)		2	5	8	13
Renal cortical thickness (mm)							
≤10	56 (77.8%)		4	18	14	20	.179
>10	16 (22.2%)		1	2	6	7
AP Diameter of pelvis in cm (mean ± SD)	6.1 ± 2		5.6 ± 1.5	6.9 ± 1.9	6.2 ± 1.6	5.7 ± 2.3	.174
Parenchymal echogenicity							
Normal	13 (18.1%)		1	4	4	4	.378
Abnormal	59 (81.9%)		24	16	16	23
Corticomedullary differentiation							
Abnormal	25 (34.7%)		0	7	8	10	.184
Normal	47 (65.3%)		5	13	12	17
Degree of hydronephrosis							
Moderate	15 (20.8%)		1	2	6	6	.266
Gross	57 (79.2%)		4	18	14	21
PCN duration (weeks)	8.1 ± 4.1		5.4±1.5	7.1 ± 3.4	8.1 ± 4.3	9.4 ± 4.3	.094
PCN output (ml/day)	757		44	200	646	1383	.000
Creatinine clearance from PCN (ccGFR) ml/min	8.3 ± 4.1		4.9 ± 1.3	4.4 ± 2.2	8.1 ± 3.8	11.9 ± 2.4	.000
Length of hospital stay (days)	2.4 ± 0.7		2.4 ± 0.5	2.7 ± 0.7	2.1 ± 0.6	2.4 ± 0.8	.08
Median follow-up (months)	13.8		2	4.5	14.7	22.1	.000
Outcome							
Reconstructive surgery	40 (55.6%)		0	2	13	25	.000
Nephrectomy	32 (44.4%)		5	18	7	2

**Table 2.** Effects of Percutaneous Nephrostomy

**Variables**	**Total**	**Group I (Split Renal Function 1-5%)**	**Group II-(Split Renal Function 5-10%)**	**Group III (Split Renal Function 11-15%)**	**Group IV (Split Renal Function 16-20%)**	*P*
Split renal function (%)						
Improved	51(70.8%)	0	10	14	27	.000
Not improved	21(29.2%)	5	10	6	0
GFR						
Improved	51 (70.8%)	0	10	14	27	.000
Not improved	21 (29.2%)	5	10	6	0

## References

[1] Zhang S Zhang Q Ji C et al. Improved split renal function after percutaneous nephrostomy in young adults with severe hydronephrosis due to ureteropelvic junction obstruction. J Urol. 2015;193(1):191 195. 10.1016/j.juro.2014.07.005) 25014578

[2] Chandra M Bachches N Bhattacharya A Attri A . The evaluation of safety and efficacy of percutaneous nephrostomy in young adult patients with severe hydronephrosis due to uretropelvic junction obstructions. Int J Res Med Sci. 2019;7(9):3260 3265. 10.18203/2320-6012.ijrms20193613)

[3] Patil SR Pawar PW Savalia AJ Mundhe ST Narwade SS Tamhankar AS . Role of calculated glomerular filtration rate using percutaneous nephrostomy creatinine clearance in the era of radionuclide scintigraphy. Urol Ann. 2017;9(1):61-67. 10.4103/0974-7796.198884) PMC5308041 28216932

[4] Nayyar R Yadav S Singh P Kumar R Seth A Dogra PN . Outcomes of pyeloplasty in very poorly functioning kidneys: examining the myths. Urology. 2016;92:132 135. 10.1016/j.urology.2016.02.045) 26970450

[5] Gupta DK Chandrasekharam VV Srinivas M Bajpai M . Percutaneous nephrostomy in children with ureteropelvic junction obstruction and poor renal function. Urology. 2001;57(3):547 550. 10.1016/s0090-4295(00)01046-3) 11248637

[6] Department of Radiodiagnosis, All India Institute of Medical Sciences, Bhuvaneshwar, India, Md SN. Dual Technique Percutaneous Nephrostomy: Experience from a Tertiary Care Centre. J Med Sci Clin Res. 2018;6(2). Available at: http://jmscr.igmpublication.org/v6-i2/185%20jmscr.pdf.

[7] Prasad N Rangaswamy D Patel M et al. Long-term outcomes in children on chronic continuous ambulatory peritoneal dialysis: a retrospective cohort study from a developing country. Pediatr Nephrol. 2019;34(11):2389 2397. 10.1007/s00467-019-04311-w) 31468143

[8] Gates GF Glomerular filtration rate: estimation from fractional renal accumulation of 99mTc-DTPA (stannous). AJR Am J Roentgenol. 1982;138(3):565 570. 10.2214/ajr.138.3.565) 7039273

[9] Taylor AT Brandon DC de Palma D et al. SNMMI procedure standard/EANM practice guideline for diuretic renal scintigraphy in adults with suspected upper urinary tract obstruction 1.0. Semin Nucl Med. 2018;48(4):377 390. 10.1053/j.semnuclmed.2018.02.010) 29852947 PMC6020824

[10] Blaufox MD De Palma D Taylor A et al. The SNMMI and EANM practice guideline for renal scintigraphy in adults. Eur J Nucl Med Mol Imaging. 2018;45(12):2218 2228. 10.1007/s00259-018-4129-6) 30167801

[11] Kabasakal L Turoğlu HT Onsel C et al. Clinical comparison of technetium-99m-EC, technetium-99m-MAG3 and iodine-131-OIH in renal disorders. J Nucl Med. 1995;36(2):224 228. 7830118

[12] Dalal R Bruss ZS Sehdev JS . Physiology, renal blood flow and filtration. In: StatPearls [Internet]. Treasure Island (FL): StatPearls Publishing; 2021. Available at: http://www.ncbi.nlm.nih.gov/books/NBK482248/. Accessed May 25, 2021. 29489242

[13] Kabasakal L Technetium-99m ethylene dicysteine: a new renal tubular function agent. Eur J Nucl Med. 2000;27(3):351 357. 10.1007/s002590050045) 10774890

[14] Gnech M Berrettini A Lopes RI et al. Pyeloplasty vs. nephrectomy for ureteropelvic junction obstruction in poorly functioning kidneys (differential renal function <20%): a multicentric study. J Pediatr Urol. 2019;15(5):553.e1 553.e8. 10.1016/j.jpurol.2019.05.032) 31277930

[15] Shokeir AA Provoost AP Nijman RJ . Recoverability of renal function after relief of chronic partial upper urinary tract obstruction. BJU Int. 1999;83(1):11 17. 10.1046/j.1464-410x.1999.00889.x) 10233446

[16] Weltings S Schout BMA Roshani H Kamphuis GM Pelger RCM . Lessons from literature: nephrostomy Versus double J ureteral catheterization in patients with obstructive urolithiasis-which method is superior? J Endourol. 2019;33(10):777 786. 10.1089/end.2019.0309) 31250680

[17] Fernández-Cacho LM Ayesa-Arriola R . Quality of life, pain and anxiety in patients with nephrostomy tubes. Rev Lat Am Enfermagem. 2019;27:e3191. 10.1590/1518-8345.3039.3191) PMC6781322 31596421

[18] Kalra S Mehra K Muruganandham K et al. Does diversion in poorly functioning obstructed kidneys in adults favors reconstructive surgeries over ablative procedures? A prospective study. Cureus. 2020;12(8):e10124. 10.7759/cureus.10124) PMC7523748 33005538

[19] Sharma U Yadav SS Tomar V . Factors influencing recoverability of renal function after urinary diversion through percutaneous nephrostomy. Urol Ann. 2015;7(4):499 503. 10.4103/0974-7796.157960) 26692673 PMC4660704

[20] Man DWK Hendry GMA Hamdy MH . Percutaneous nephrostomy in pelviureteric junction obstruction in children. Br J Urol. 1983;55(4):356 360. 10.1111/j.1464-410x.1983.tb03321.x) 6883041

[21] Wagner M Mayr J Häcker FM . Improvement of renal split function in hydronephrosis with less than 10 % function. Eur J Pediatr Surg Off J Austrian Assoc Pediatr Surg Al Z Kinderchir. 2008;18(3):156 159. 10.1055/s-2008-1038445) 18484518

[22] Ransley PG Dhillon HK Gordon I Duffy PG Dillon MJ Barratt TM . The postnatal management of hydronephrosis diagnosed by prenatal ultrasound. J Urol. 1990;144(2 Pt 2):584 587; discussion 593-594. 10.1016/s0022-5347(17)39528-9) 2197441

[23] Dhillon HK Prenatally diagnosed hydronephrosis: the Great Ormond Street experience. Br J Urol. 1998;81(suppl 2):39 44. 10.1046/j.1464-410x.1998.0810s2039.x) 9602794

[24] Pode D Shapiro A Gordon R Lebensart P . Percutaneous nephrostomy for assessment of functional recovery of obstructed kidneys. Urology. 1982;19(5):482 485. 10.1016/0090-4295(82)90603-3) 7080320

